# Extra Supplement—The Nursing Mirror

**Published:** 1890-05-10

**Authors:** 


*The Hospital*, May 10, 1890. *Extra Supplement.*

### "The Hospital" Nursing Mirror.

Being the Extra Nursing Supplement of "The Hospital" Newspaper.

All Contributions for this Supplement should be addressed to the Editor, The Hospital, 140, Strand, London, W.C., and should have the word "Nursing" plainly written in left-hand top corner of the envelope.

### En Passant.

**Dramatic Performance.**—The Silurian Dramatic Club gave a delightful performance at Harrington Hall last week in aid of the Nurses' Club, 12, Buckingham Street. The Nurses' Club is badly in need of books, and medical books are often very expensive, hence the above performance, which it is hoped will benefit the club to the extent of £10. The acting was very good at Harrington Hall, Mrs. Nichol as Mrs. Malaprop perhaps carrying off the palm. The orchestra was also excellent, and all the arrangements were well carried out.

**Redditch Nursing Association.** — The first annual meeting of this association was lately held, when the work done by the one nurse was highly commended, and the necessity for a second nurse was forcibly expressed. Sir Harry Verney kindly addressed the meeting, and moved the following resolution: "That this meeting views with great satisfaction the result of the nurse's work in the town for the past year, and pledges itself to use the utmost efforts to raise the funds necessary for the continuance of the work, and, if possible, for the support of two nurses." Funds are satisfactory for the present, but annual subscribers are badly needed.

**District Nursing at Ballymena.**—The report of the District Nursing Society of Ballymena has just been published. The statistics ought to speak for themselves. The number of cases on the list, April 1, 1889, was 25; do., registered during the year, 213; total, 238. These include—cases of consumption, 23; burns, 34; and pneumonia and bronchitis, 54. The number of visits paid by the nurses during the year was 8,397. Several patients were attended at night as well as day. In urgent cases, the nurses were several consecutive hours with the patient, staying with them to 10 or 12 p.m. The nourishment supplied from kitchen has been—pints of milk, 10,808; dozens of eggs, 212; lbs. of meat, 339; puddings, 159; fruit preserves, wine, cold meat, fish, bread, vegetables, tea, sugar, and other groceries. There are two nurses at work, and during the seven years the society has existed its total income has been £1,721.

**Nurses and Infection.**—Dr. Atkinson, of Surbiton, proposes the following precautions for private nurses who go to infectious cases: "Every nurse should be provided with two tin boxes, and each box should contain a complete change of clothing. One of these, with a permanent distinctive mark upon it, should be placed in a spare room quite unconnected with the infected ones, directly it enters the house; the other, of course, should go to the room which the nurse is to occupy during the continuance of the illness. In this way the nurse, after leaving all her infected clothing behind the sheet in a solution of creolin or carbolic acid, can, after her bath and wrapping herself in a clean blanket which has been thrown down outside the sheet, go into the uninfected room and dress in perfectly fresh clothing. The management of the infected room has been perfectly dealt with before, and, therefore, there is no occasion to enter upon this matter. Each nurse should be supplied with a set of rules, showing what precautions are necessary. Going back to the infected rooms after they have been once left should be strictly forbidden, at any rate till they have been properly dealt with."

**Short Items.**—Some successful tableaux have been given at Bournemouth in aid of the Victoria Home for Nurses.—Miss Close, of Newcastle, has lately recovered from an attack of diphtheria contracted in the discharge of her duties.—Two Sisters of Charity nursed the Shah's wife, who has lately undergone an operation on the eyes.—Miss Morse, matron of the Children's Hospital at Nottingham, has considerably increased the income of the institution by arousing the public interest.—Miss Maud Lobb is leaving the Sydney Hospital for Children, and returning to England next autumn.—We are glad to hear that the Camborne District Nurses' Fund has been so successfully worked that a second nurse is to be engaged.

**Liverpool Nurses.**—The *Liverpool Mercury* for April 28th contained a long and interesting article on "The Rise and Progress of the Nurses' Home." It was in 1862 that the idea was first mooted in Liverpool, and now the Dover Street Home houses sixty nurses. There are also homes for district nurses in Shaw Street and Upper Parliament Street. A lady has come forward and given £1,500 for the foundation of a nurses' home at the north end. "The twentieth district will thus be born into the world, and probably we shall see no fuss made about it. When an ironclad is launched or a baby king is sick we hear of every detail, but when a nurses' home is opened the world knoweth it not. Yet the latter is not the least important event. But the blind world moans in its agony and dreams that kings and ironclads are helpers on the path of universal peace."

**Belfast Nurses.**—The annual meeting of the Society for Providing Nurses for the Sick Poor of Belfast was held on April 30th, Mr. Ewart in the chair. We quote the following from the report: "During the late sad and unhealthy time some of our nurses were much overworked, as in nearly every family where they were attending several members seemed to be ill at once; but we are thankful to say they all escaped without serious illness. Believing that underfeeding, which is slow starvation, is at the root of many of the ailments we meet with, we have, whenever the sick are unable to procure it for themselves, given as much nourishment as our funds would admit. We are convinced that milk is 'one of the best medicines in the Pharmacopœia or out of it,' and have therefore spent £385 17s. 1d. on new milk. We have paid £63 for meat, and £42 for eggs. Amongst our 934 patients we have lent 1,224 articles, waterbeds, cushions and chairs. Some 1,800 articles, contributed by 609 associates of the Needlework Guild, besides a great deal of half-worn clothing, have been distributed. The nurses have paid 30,061 visits. 424 of the patients were sent direct to us by the doctors attending them, and many were sent from the different hospitals. This actual work which can be set down in figures is sufficiently important. It means the lifting of heavy burdens, and represents much alleviation of suffering. But the indirect influence of our society is greater still and more far-reaching. In addition to the nine nurses employed, we have twenty-three ladies visiting in the districts and assisting them in various ways. Two of these ladies have had hospital training, and gives us invaluable help, even taking a nurse's place occasionally, during her illness or holiday time. By the kindness of the committee of the Nurses' Home, six of our nurses were enabled to attend medical lectures, given by Dr. Calwell and Dr. Strafford Smith."

### Nursing Lectures.

By Percy Lewis, M.D.

### Lecture XI.—NERVOUS DISEASES.

The brain is the seat of consciousness, and is the organ by which the rest of the body is governed. It consists of innumerable little masses of nervous matter called nerve, centres aggregated together, each little mass having some definite function to perform, and connected with the part over which it exercises its influence by long strands of nervous tissue known as nerves. Some nerves convey messages from the brain to the muscles and make them contract, other nerves bring messages of feeling sense, or pain to the brain from the skin or other organs. Without nerves connecting the brain with the rest of the body there could be no feeling or motion; without the brain there could be neither feeling, motion, nor consciousness. It is because of the great importance of the brain that it is enclosed inside the bony box called the skull. The brain is continued from the skull into the spine as the spinal cord. This consists for the most part of nerve fibres passing from the brain to the rest of the body and from the rest of the body to the brain, but it also contains separate centres of its own, controlling definite acts.

If a nerve going to a limb is cut or destroyed by tumours, the limb cannot move or feel; it is said to be paralysed. But it may be paralysed in another way; the centre for motion may be destroyed by tumours, injury or hæmorrhage into it (apoplexy); then, though able to feel, it will not be able to move; or its centre for feeling may be similarly affected, when, though able to move, it will not be able to feel. In the latter case it is said to be anæsthetic. All the different movements and functions of the body are controlled by different nerve centres; the most important to life, viz., those controlling the movements of the heart, respiration and swallowing, being grouped together in the prolongation of the spinal cord into the part of the brain known as the medulla. Roughly speaking the brain is divided into two halves, each half controlling the opposite side of the body. When the motor centres on one side of the brain, say the right, get cut off by blocking or rupture of blood vessels, the left side of the body gets paralysed, and the patient is said to have "hemiplegia." Paraplegia is when both legs get paralysed from some disease or injury of the spine.

The brain and spinal cord are covered over by a bag of membranes containing fluid, so that they rest as it were on a water cushion, which thus to a great extent protects them from shock and jars. Inflammation of these membranes is called meningitis. Centres for different movements, as for the arm or leg, may get separately inflamed, giving rise to infantile paralysis, the cause of so many wasted limbs in children. Paralysis may be induced by various poisons as lead, mercury, or alcohol.

The symptoms of special importance in nervous disease are paralysis, involuntary movements, rigidity, difficulty in swallowing or in speaking, neuralgic and other pains, headache, vomiting, sleeplessness, drowsiness, illusions, delirium, fits, each of which we will now discuss. Paralysis may be the symptom for which the patient is admitted, or it may come on gradually or suddenly while he is in the hospital. When a case is admitted for paralysis, endeavour to note how much he is paralysed, with special reference to particular movements, such as buttoning his clothes or picking up pins; thus you will more readily recognise any extension of it. Hysterical patients get various forms of paralysis which frequently change; first it may be affecting one leg and then one arm, or sensation may be implicated in the same manner. A common form of paralysis is that of the nerve supplying the face muscles of expression. The symptoms of facial paralysis when well-marked are very characteristic; still the degree of paralysis varies considerably. In well-marked cases, there is a total loss of expression and movement on the paralysed side, the mouth becomes drawn to the sound side when the patient speaks; on eating, food gets between the gums and the cheek; and he is unable to shut the eye on that side. Squint is due to paralysis of some of the muscles which move the eyeball; this consequently becomes drawn out of its normal direction by the unopposed sound ones.

Closely allied to paralysis are involuntary movements. Starting at night directly a patient gets to sleep may be very distressing if it is constantly waking him up. You are all of you acquainted with the involuntary movements which occur in chorea, or St. Vitus' dance, when the patient attempts to do anything. Twitching of muscles, especially of face, trembling of limbs, shaking or nodding of the head, all occur as involuntary movements in different diseases. Perhaps the most striking involuntary movements are those which occur in tetanus. This is a painful spasmodic disease affecting at first small groups of muscles, as those of the hand, fore-arm, or jaw (lockjaw), the muscles either remaining continually contracted or else the contractions become interrupted with periods of relaxation. Later on, if not relieved, nearly all the muscles of the body get affected; the spasms, if not continuous, become easily excited by very slight causes, such as moving the bed-clothes or shutting the door. The contractions may become so general and strong that the patient during the spasm rests on his heels and the back of his head only. The necessity for avoiding all movement and noise is in this case obvious.

In some nervous diseases a condition known as rigidity comes on. An arm or a leg becomes as though made of wood, refusing to move or bend at the joints.

In a form of paralysis call Bulbar paralysis, the patient gradually loses the power of speech and of swallowing; at first very slight, the paralysis may become so great that speech is unintelligible and swallowing almost impossible. These cases have to be fed with great care with liquids in small quantities only. Solids must not be given on account of their great liability to get into the larynx and by cutting off the air, suffocate, and so cause the death of the patient.

Various characteristic pains occur in nervous diseases; thus lightening pains in legs and sensations of cords round the waist appear in locomotor ataxy; pain in the throat and on the top of the head in hysteria. Headache has so many causes of origin that I will not attempt to discuss it. Vomiting is always an important symptom in nervous disease, occurring in meningitis, locomotor ataxy, and many others.

### The Private Nurses' Co-operation.

A large number of private nurses at present working in the metropolis on their own account have determined to combine together in a mutual association. A provisional committee has been formed, which is at present working out the details of the scheme, and owing to the generous help of a non-professional friend, £200 has been banked to the credit of the co-operation. The object of the society, shortly stated, is "to supply private nurses, thoroughly trained and certificated, to practitioners and the public, through the agency of nurses who have combined for their mutual benefit and welfare," and the distinctive features are: (1) To establish a central place of reference, where doctors, patients, and nurses may readily obtain the services of a competent private nurse at short notice; (2) to prevent any risk of infection to patients by providing the adequate accommodation under proper supervision for the private nurse when she is not engaged upon a case; (3) to place the Private Nurses' Co-operation under the management of a representative committee in conjunction with patrons and patronesses, who from their position and influence must materially aid the nurses in their efforts to help themselves; and (4) generally to permit the nurses to take part in the management of the co-operation, to support such co-operation by a percentage of the nurse's earnings sufficient only to meet the working expenses; to provide that each nurse shall be assigned such cases only as may be suitable to her individual capabilities, and to do everything necessary to meet the requirements of the medical profession and of the public. The movement is one to which we are very glad to give publicity, and which we are sure will meet with success if under proper management. We are glad to hear that a great number of worthy and weighty names have been given in support of this society, and that the medical profession is proving itself most just and generous in supporting the nurses in their effort to combine for their mutual welfare. Part of the scheme consists not only in supplying the nurses with a central office, but in supplying the nurses with lectures and clinical instruction, so that their knowledge may never grow rusty; and in trying in every way to give these women, who are now more or less isolated and therefore at a disadvantage, the benefits which nurses connected with hospitals have ever at their disposal. There can be no doubt that the opening of institutions in connection with hospitals is a step in the march of progress, but at present the hospitals cannot train their nurses fast enough to supply their private staff, and there is no reason why in the meanwhile nurses already trained, but severed by time from their hospitals, should not combine to reap such advantages as mutual help can give. Again, some nurses prefer the stimulus of working up their own connection, rather than the peace of a fixed salary. These venturesome spirits join the co-operation at their own risk, and work the better simply because of the risk. The rules and regulations of the Nurses' Co-operation are to be modelled on those of the American Directory for Nurses: strict inquiry will be made into the training and character of every nurse who joins; and power will be placed in the hands of a trained lady superintendent to direct and control the nurses. The Co-operation is to be no Liberty Hall, no refuge for the rebellious and cantankerous, but as trained nurses will be represented on the committee the rules will be just and suitable; and such as they are, they must be strictly kept by all members. Every true nurse knows that without order and discipline no combination can ever be successful.

### Everybody's Opinion.

[*Correspondence on all subjects is invited, but we cannot in any way be responsible for the opinions expressed by our correspondents. No communications can be entertained if the name and address of the correspondent is not given, or unless one side of the paper only be written on.*]

### THE JUNIUS S. MORGAN MEMORIAL.

A Private Nurse writes: It was with much regret I read the announcement of Mr. Junius Morgan's death, and feel what a great loss it will be to us nurses and many others. I shall be only too glad to do my part by collecting the sum of £5 towards the Benevolent Branch of the Pension Fund, and for a memorial to one who did so much for us nurses. I hope all other nurses will try their best to do the same, only adding my earnest hope that the amount raised will be entirely used for the benefit of needy nurses.

Nurse Wilson writes: I am delighted to inform you that my £5 is now raised. Our matron kindly allowed me time to go round to a good number of friends, and I was very successful, with of course a few disagreeable experiences.

Nurse Black writes: May I suggest that every nurse should commence her collecting among her fellow-nurses, and be sure and put in what she herself can afford? I have done this with the greatest success; every nurse can give a trifle, and trifles soon mount up, and then with the kind aid of our friends outside the profession it is easy to raise the £5.

### UNIFORMS FOR JUNE.

A Cornishwoman writes: May I suggest, as a person deeply interested in the noble band of women nursing in various departments who are not wearing outdoor uniform, that the uniform for nurses who may be presented to her Royal Highness the Princess of Wales to receive their certificates of honour, should be a navy blue cashmere, or thin cloth, "Russian" cloak, the fronts lined with silk; navy blue "Princess" bonnet trimmed with navy velvet; strings to match with white strings (sarcenet) over; no veils. This costume would be universally becoming, as it is a style suited to all figures.

Nurse Rose advises "An Interested One" to devise for herself a pretty nurse's dress and wear it always, only taking care she does not tread on the toes of any hospital or institute in her locality by not making it sufficiently distinct from theirs. She will find innumerable advantages in so doing, and be saved for ever the harrassing question, "What shall I wear?"

E. C. read two suggestions from "S." in last week's Hospital, and, in reply, ventures to suggest, first, that we take it for granted that we shall wear outdoor uniform at the presentation, as otherwise we should be giving a great deal of unnecessary trouble at Marlborough House. Secondly, all nurses attached to hospitals and nursing institutions will wear their own uniform at the presentation, and that private nurses who wear no uniform should, if willing, wear a uniform after this description: Grey alpaca dresses, black alpaca cloaks or cashmere, whichever is liked best, black bonnets trimmed with velvet, black veils, and white muslin strings. If this suggestion is accepted I will gladly forward to office of your paper a yard of alpaca the colour I would suggest, so that patterns may be had by any nurses who wish.

"S. E. A." writes: May I, another private nurse, endorse the suggestions of "S." in last week's Hospital. As our hospitals and institutions will be no doubt well represented, could not we, too, wear a dress of a pretty washing material very similar in colour. For instance, let us wear grey linen and a pale pink or blue. Grey linen looks best but washes so badly. I think the effect would be much prettier than each private nurse appearing in our odd colour; it might also prevent us from trespassing upon the uniform of any other institution.

### The "First Thousand."

One by one, one by one,
Lo! they come onward;
Joining the Pension Fund,
Come the Ten Hundred!
"Sick, ye shall have our aid;
Old, have a pension paid."
This had the founders said—
Heard that Ten Hundred!

"Pay us your fees," they said,
Was there a nurse afraid?
Not though for all she knew
Some might have blundered!
Bravely they made reply,
"We may be sick, or die;
Trust them, nor reason why,
We'll join the Pension Fund"—
Thus the Ten Hundred!

Warnings to right of them,
Warnings to left of them,
Warnings in front of them
Volleyed and thundered!
Heedless of all they hear,
Whispers of doubt and fear,
Straight to the office come
All the Ten Hundred!

Opened their purses there,
Leaving them often bare!
But still they do and dare,
Helping their comrades, while
All their world wondered.

Bravely they paid their fees,
Though none the future sees.
Laggard and coward
Presently follow these—
Long they had pondered;
Counted they cannot be,
With the Ten Hundred!

Honours to right of them,
Honours to left of them,
Honours all round them,
Met the Ten Hundred!
Loudly the press extols;
Fame every name enrols;
Princess presents the scrolls
To that Ten Hundred!
Who, in the teeth of scorn,
Led on the hope forlorn,
Manning a vessel that
Else might have foundered.

Oh! the brave show they made!
In their quiet garb arrayed,
From the world sundered.
When from the royal hand
They took the scroll that said—
"One of the Thousand!"
Honour the Nurse Brigade!
Oh! the brave show they made!
Noble Ten Hundred!

S. Ruth Canton.

### Examination Questions.

Over twenty answers were received to the last examination question, those from Oxford, Bristol, and Wandsworth being the best. It was extremely hard to judge; the question being so exact, the answers were short and concise, and bore a strong resemblance to one another. After consideration, however, we have awarded the prize to Nurse Colborne, to whom we have sent Dr. Mackenzie's "Home Medicine and Surgery," and whose answer will be printed next week. The question for May is, "What Precautions Should a Nurse in Charge of a Diphtheria Case Observe?" Answers must reach our office by May 20th, and we hope they will be numerous, for this will be the last competition till the autumn once more brings us long, dark evenings.

### Presentations.

Victoria Hospital, Guernsey.—Miss Alice Tourtel, who has lately resigned her post of matron to this Cottage Hospital owing to ill-health, has been presented by the subscribers with a handsome silver Queen Anne tea service, enclosed in a morocco case, comprising sugar basin, milk jug, and six spoons, and a tea-pot, the latter article bearing the following inscription: "Presented to Miss Alice Tourtel, by the Subscribers to the Victoria Cottage Hospital, Guernsey, 1st May, 1890." Miss Tourtel was much touched by this pleasant recognition of her services.

The Russian Red Cross.—Miss Kate Marsden, one of the five English sisters who went to Bulgaria to attend to the Russian sick and wounded in the campaign of 1878, has been received by the Russian Empress at Gatchina with much gracious amiability, and has been awarded the insignia of the Russian Red Cross. Miss Marsden has also received the Imperial permission to visit all the hospitals in the Empire, and she intends making a tour in order to see such lepers as are to be found in a country where leprosy, although known to be very prevalent by one or two specialists, has hitherto been almost totally ignored by the authorities, and has not yet been properly investigated or subjected to any organised method of treatment. Therefore, Miss Marsden's more or less accidental creation of an official interest in leprosy in Russia may lead to important results.

### Appointments.

Bristol General Hospital.—Miss A. L. Cox has been appointed assistant matron to the above hospital. Miss Cox trained at the Derby Infirmary, where she remained five years, afterwards joining the Kent Nursing Institution. For the last six months Miss Cox has been acting as night superintendent at Bristol, and having won golden opinions there, she has been naturally appointed to the higher post.

Rochdale Infirmary.—Miss Alice Chapman, who trained at Manchester Royal, and has since worked at Aylesbury and Oldham, has been appointed matron of the Rochdale Infirmary.

### Notes and Queries.

To Correspondents.—1. Questions may be written on post-cards. 2. Advertisements in disguise are inadmissible. 3. In answering a query please quote the number. 4. If a private answer is desired, a stamped addressed envelope must be forwarded.

Notice.—We must really ask our correspondents to be more particular in enclosing name and address. Constantly we receive queries or letters with no name attached.

### Queries.

(8) *Home Wanted.*—In London, for an epileptic girl. Parents could pay small sum.—*Doris.*

(9) *Stays.*—Can anyone tell me where to get suitable stays for a patient suffering from tumour of the breast?—*Nurse Gertrude.*

(10) *Free Passage to Australia.*—Can anyone tell me how a trained nurse could earn her passage out to Australia?—*Sister Mary.*

### Answers.

*Nurse Agnes.*—(1) Yes. (2) We hope to publish a paper on the subject sometime, but had you not better buy a cheap book on nursing, and study up these subjects for yourself?

*Probationer.*—It is impossible to say how long you may have to wait for a vacancy; it may be months or years.

(50) *A Country District Nurse.*—The address of the Boot Company here is Newborough, Scarborough; they have shops in all big towns. The boots may be uncomfortable at first, if you have naturally hot feet; I have not. I do not find that the rough roads cut them, and you can have nails in them if you like.—*Sister Dora.*

*Automatic Respirator.*—No London or other agent is mentioned for the sale of the automatic respirator.

(9) *Stays.*—Apply to Madame Wood, 131, Edgware Road, W.; she makes special corsets to suit such cases for a guinea.

*One who Could not Afford £5.*—We hope the plan will not encourage improvidence. It has been so warmly taken up, it is sure to be successful; and the nurses themselves are giving to the best of their abiiity.

*F. L. E.*—We will publish your scheme later, when the present one is accomplished, as it will be, in spite of your fears, by June.

### Vacancies.

The following vacancies are announced:

### Matrons.

Arbroath Infirmary.

Folkestone Victoria Hospital; £50.

Tewkesbury Hospital; £45.

Woodilee Asylum, Lenzie; £80.

### Sisters.

Bolton Infirmary; £25.

Cardiff Infirmary; £28.

Glasgow Victoria Hospital; £24.

Monsall Fever Hospital; £32.

Newcastle Royal Infirmary; £30.

### Nurses.

Bangor Nursing Institute; £25.

Birkenhead Boro' Hospital; £25.

Birmingham Homœopathic Hospital; £20.

Blackburn Infirmary; £27.

Blackheath Nursing Institution.

Brixton Institute.

Carlisle Home for Incurables; £25.

Carmarthen Infirmary; £23.

Central London Throat and Ear Hospital; £14.

Chelsea Infirmary; £18.

Chester Deaconess' House; £24.

Children's Hospital, Pendlebury; £20.

Croydon Hospital; £25.

East London Nursing Society.

Fleming Memorial Hospital; £28.

Fulham Union; £20.

General Nursing Institute.

Glasgow Sick Poor Association; £25.

Greenwich Union; £27.

Hanwell Ophthalmic School; £26.

Isle of Wight Royal Infirmary; £20.

Jersey Nurses' Institute; £25.

Kendal Hospital; £28.

London Temperance Hospital (night); £25.

Longton College Hospital.

Mr. Wilson's Institution.

Newcastle Royal Infirmary; £20.

Newlands, Ryde.

North-West London Hospital; £2 per month.

Southport Infirmary; £25.

St. Helena Home.

St. Helen's Cottage Hospital.

St. Mark's Hospital; £25.

Stratford-on-Avon Hospital; £20.

St. Saviour's Infirmary; £20.

Wallasey Fever Hospital; £25.

Weston-super-Mare (night); £25.

Workhouse Infirmary Nursing Association.

### District Nurses.

Camborne, Cornwall.

Cambridge Home for Nurses.

Gloucester District Society; £25.

Jersey Institute; £25.

Worcester County Institution.

### Probationers.

Croydon Union; £10.

Gateshead Hospital for Sick Children.

Buckinghamshire General Infirmary.

Grimsby Hospital.

Gorleston Cottage Hospital.

H.M. Hospital, Stepney.

Leeds Nurses' Institution; £14.

Monsall Fever Hospital; £15.

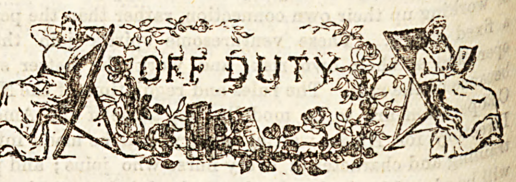

### Amusements and Relaxation.

### N.B.—New Word Competition Commenced April 5th, 1890, ends June 28th, 1890.

Three prizes of 15s., 10s., 5s., will be given for the largest number of words derived from the words set for dissection.

Proper names, abbreviations, foreign words, words of less than four letters, and repetitions are barred; plurals, and past and present participles of verbs, are allowed. Nuttall's *Standard* dictionary only to be used.

N.B.—Word dissections must be sent in WEEKLY to 140, Strand, W.C., arranged alphabetically, with correct total affixed.

The word for dissection for this, the SIXTH week of the quarter, being

"HAWTHORN."

| Names. | May 1st. | Totals. | Names. | May 1st. | Totals. |
|---|---|---|---|---|---|
| Adeline | 54 | 149 | Agamemnon | 56 | 171 |
| Patience | 58 | 176 | G. E. J. C. | — | 34 |
| Lightowlers | 56 | 167 | S. E. A. | — | 24 |
| Canary | — | 86 | Jenny Wren | 51 | 165 |
| M. E. S. | — | 37 | Multum in parvo | — | 32 |
| Ambition | — | 36 | Ecila | — | 39 |
| M. W. | 56 | 170 | Weta | 53 | 131 |
| Esperance | 57 | 171 | Henri | 54 | 149 |
| Judy | — | 37 | Holland | 56 | 171 |
| Reldas | 58 | 173 | T. J. | 33 | 111 |
| Merenda | — | 29 | Nurse Isabel | — | 17 |
| Lucy Locket | — | 40 | Nurse Faith | — | 9 |
| Camellia | — | 30 | E. S. Z. | — | 10 |
| Qu'appelle | 57 | 168 | G. P. | — | 10 |
| Tinie | 58 | 177 | Stumps | — | 33 |
| Nurse Mildred | 40 | 127 | Bolton | — | 8 |
| Coralie | 57 | 173 | Jesmur | 53 | 53 |
| Gladys | 56 | 172 | | | |

### Notice to Correspondents.

N.B.—Each paper must be signed by the author with his or her real name and address. A *nom de plume* may be added if the writer does not desire to be referred to by us by his real name. In the case of all prize-winners, however, the real name and address will be published.

Competitors can enter for *all* quarterly competitions, but no competitor may take more than *one first prize* during the year.

